# NLRP3 Inflammasome is Activated in Rat Pancreatic Islets by Transplantation and Hypoxia

**DOI:** 10.1038/s41598-020-64054-9

**Published:** 2020-04-24

**Authors:** Vanessa Lavallard, David Cottet-Dumoulin, Charles-Henri Wassmer, Caroline Rouget, Géraldine Parnaud, Estelle Brioudes, Fanny Lebreton, Kevin Bellofatto, Ekaterine Berishvili, Thierry Berney, Domenico Bosco

**Affiliations:** Cell Isolation and Transplantation Center, Department of Surgery, Faculty Diabetes Center, Geneva University, Hospitals and University of Geneva, Geneva, Switzerland

**Keywords:** Apoptosis, Type 1 diabetes

## Abstract

Hypoxia, IL-1β production and oxidative stress are involved in islet graft dysfunction and destruction. However, the link between these events has not yet been determined in transplanted islets. The goal of this study was to determine whether NLRP3 inflammasome is responsible for IL-1β production and if it is activated by hypoxia-induced oxidative stress in transplanted islets. Rat islets were transplanted under the kidney capsule of immunodeficient mice. At different times post-transplantation, blood samples were collected and islet grafts harvested. Rat islets were also incubated *in vitro* either under normoxia or hypoxia for 24 h, in the absence or presence of inhibitors of NLRP3 inflammasome (CASP1 inhibitor) or oxidative stress (NAC). *NLRP3, CASP1*, *IL1B*, *BBC3* pro-apoptotic and *BCL2* anti-apoptotic genes in transplanted and *in vitro* incubated islets were then studied using real time PCR. IL-1β released in the blood and in the supernatant was quantified by ELISA. Cell death was analysed by propidium iodide and Annexin-V staining. *NLRP3*, *CASP1* and *BBC3* in transplanted rat islets and IL-1β in blood transiently increased during the first days after transplantation. In islets incubated under hypoxia, *NRLP3, IL1B and CASP1* and IL-1β released in supernatant increased compared to islets incubated under normoxia. These effects were prevented by the inhibition of NLRP3 inflammasome by CASP1 or oxidative stress by NAC. However, these inhibitors did not prevent hypoxia-induced rat islet death. These data show that NLRP3 inflammasome in rat islets is transiently activated after their transplantation and induced through oxidative stress *in vitro*. However, NRLP3 inflammasome inhibition does not protect islet cells against hypoxia.

## Introduction

Pancreatic islet transplantation is a non-invasive method and a promising therapy for type 1 diabetic patients. Since the publication of the Edmonton protocol in 2000^1^, improved islet isolation procedures and better adapted immunosuppressive treatments enabled maintaining insulin-independence in 50% of the grafted patients after 5 years^2^. Despite these encouraging results, more effort is required before success rates of islet transplantation becomes similar to those of pancreas transplantation. A major concern is that isolated islets are submitted to different hypoxic events before and after transplantation, which negatively impact their function and viability^3–5^. The aortic clamping, which precedes organ harvesting, already induces pancreatic tissue ischemia. Additionally, the islet isolation procedure implicates disruption of vascularization, and isolated islets are often maintained in culture for days before transplantation. Under these conditions, the centrally located cells in islets are poorly nourished and oxygenated. Insufficient oxygenation of islets continues after transplantation since neovascularization of islets is not considered complete until after a couple of weeks duration. Therefore, islets undergo hypoxic conditions for several weeks^6–8^ after aortic clamping, which contribute to suboptimal engraftment and graft function. The adverse effects of hypoxia on islets could be prevented in future by pharmacological agents or other strategies that target cellular pathways activated by hypoxia. However, until then, it is essential to better understand the molecular pathways activated in response to hypoxia. Hypoxia-inducible factor (HIF) regulates a broad array of genes during hypoxic events and could be a key player in the phenomena occurring at the time of islet engraftment. HIF is a heterodimer protein composed of one O_2_-dependent subunit (HIF-1α) and a regulatory subunit (HIF-1β). In normoxic conditions, HIF-1α is degraded by the proteasome. Under hypoxia, HIF-1α is stabilized and translocates into the nucleus. There it binds to HIF-1β and activates genes involved in angiogenesis, cell proliferation/survival or glucose metabolism^9,10^. Hypoxia is also linked to oxidative stress involving the production of reactive oxygen species (ROS), which are deleterious for cell survival.

In a previous study, we showed that hypoxia activates NLRP3 (NOD-like receptor family, pyrin domain containing 3) inflammasome in human islets^11^. NLRP3 inflammasome is a multiprotein complex involved in the production of the proinflammatory cytokine interleukin-1β (IL-1β). This cytokine is involved in β-cell destruction in type 1 diabetes via the innate immune system and also plays a deleterious role in islet survival after transplantation^12–15^. Studies have shown the involvement of oxidative stress in the activation of NLRP3 inflammasome via hypoxia-induced ROS production^9,16^. However, the expression of NLRP3 inflammasome in grafted islets and its activation by ROS in islets have not yet been studied. In this study we investigate whether NLRP3 inflammasome is activated in transplanted islets and whether this effect is mediated by hypoxia-induced oxidative stress.

## Materials and methods

### Animals

Male Sprague Dawley rats (8-weeks-old) and male CB17 SCID mice (9-weeks-old) were purchased from Janvier (Le Genest St-Ile, France). All animals were kept in our local animal facilities with free access to food and water. All experiments were conducted under protocols reviewed and approved by the Geneva Institutional Animal Care and Use Committee (Direction Générale de la Santé) (License number: GE/59/17). All experiments were performed in accordance with relevant guidelines and regulations.

### Rat islet isolation and culture

Rat islets were isolated by collagenase digestion of the pancreas followed by purification on discontinuous Ficoll gradients as described previously^17,18^. Islets were incubated overnight at 37 °C in Dulbecco’s modified Eagle medium (Invitrogen, Basel, Switzerland) containing 10% fetal calf serum, 11.2 mM glucose, penicillin, and streptomycin (hereafter referred to as complete DMEM (Dulbecco’s modified Eagle medium)). Then islets were submitted *in vitro* to different treatments or transplanted to CB17 SCID mice.

### Islet treatments

Aliquots of 500 rat islet equivalent (IEQ) were incubated in 1.5 ml complete DMEM under hypoxic (1% O_2_) or normoxic conditions (21% O_2_) at 37 °C for 24 h. When required, the medium was supplemented with 50 μM Z-WEHD-FMK (R&D Systems, Inc., Minneapolis, USA), which is an inhibitor of caspase-1, or 5mM N-acetylcysteine (NAC) (Sigma, Saint-Louis, Missouri, USA), which is an inhibitor of ROS production. Islets were then analysed for cell death or stored at −20 °C in RLT buffer (Qiagen) + β-mercaptoethanol (BioRad) until RNA extraction. Culture supernatants were collected and stored at −20 °C until IL-1β measurements.

### Islet transplantation

Rat islets were transplanted under the kidney capsule of CB17 SCID mice as previously described^19^. Briefly, under anaesthesia (isoflurane), the left flank of mice was shaved and a small incision was made to expose the kidney. Five hundred rat IEQ were loaded in a PE50 polyethylene tubing (PhyMep, Paris, France) and injected under the kidney capsule using a Hamilton syringe (Reno, NV, USA). Just before transplantation and 2, 5, 7 and 14 days after transplantation, blood samples were collected and stored at −20 °C until use. At days 2, 5, 7 and 14, the graft-bearing kidneys were removed and islet grafts were harvested using microdissection instruments and stored at −20 °C in RLT buffer (Qiagen) + β-mercaptoethanol (BioRad) until RNA extraction. At d0, 500 rat islets loaded in a PE50 polyethylene tubing were immediately collected in RLT buffer + β-mercaptoethanol and stored at −20 °C. As controls, Sham-operated mice were injected with 0.9% NaCl under the kidney capsule. The kidneys were removed at the same time intervals as the transplanted mice, and kidney capsule tissue was used for RNA extraction.

### Real-time quantitative PCR analysis

RNA was extracted from frozen rat islets, islet grafts or kidney capsule tissue using the RNeasy minikit (Qiagen, Courtaboeuf, France). RNA was reverse transcribed using the High Capacity cDNA Reverse transcription kit (ThermoFischer Scientific, Waltham, MA, USA). Gene amplification was achieved with the RT-PCR method using the TaqMan Fast Advance Master Mix (ThermoFischer Scientific). Primers used for amplification were purchased from ThermoFischer Scientific: rat Actb (Rn00667869-m1), rat IL1B (Rn00580432-m1), rat NLRP3 (Rn04244625-m1), rat CASP1 (Rn00562724-m1), rat BCL2 (B-cell lymphoma 2) (Rn99999125-m1), rat TXNIP (Rn01533891-g1) and rat BBC3 (BCL2 binding component 3) also known as p53 upregulated modulator of apoptosis (PUMA) pro-apoptotic gene (Rn00597992-m1). Gene expression values were normalized based on the housekeeping gene Actb and calculated based on the comparative cycle threshold Ct method (2-ΔCt method).

### IL-1β and caspase-1 measurements

IL-1β released in culture supernatants and mouse sera was measured using a rat-specific enzyme-linked immunosorbent assay (ELISA) kit (R&D systems, Minneapolis, USA) following the manufacturer’s instructions. Caspase-1 released in culture supernatants was measured using an ELISA kit (Bio-Techne AG, Zug, Switzerland) following the manufacturer’s instructions.

### Analysis of cell death

Rat islets were dissociated into single cells by trypsinization, then washed and resuspended in binding buffer (10 mM HEPES, 0.14 mM NaCl, 2.5 mM CaCl2, pH 7.4). Apoptosis and necrosis were determined by staining with Annexin V (Biolegend, San Diego, USA) and propidium iodide (PI) (Axxora, Enzo Life Sciences, Switzerland), respectively, according to manufacturer’s instructions. Cell fluorescence was analysed with an Accuri flow cytometer.

### Statistical analysis

Differences between means were assessed either by the Student’s *t*-test or by 1-way ANOVA. Where ANOVA was applied, Tukey or Dunnett post-hoc analysis was used to identify significant differences between groups.

## Results

### NLRP3 inflammasome is transiently activated in transplanted islets

**Figure 1:**
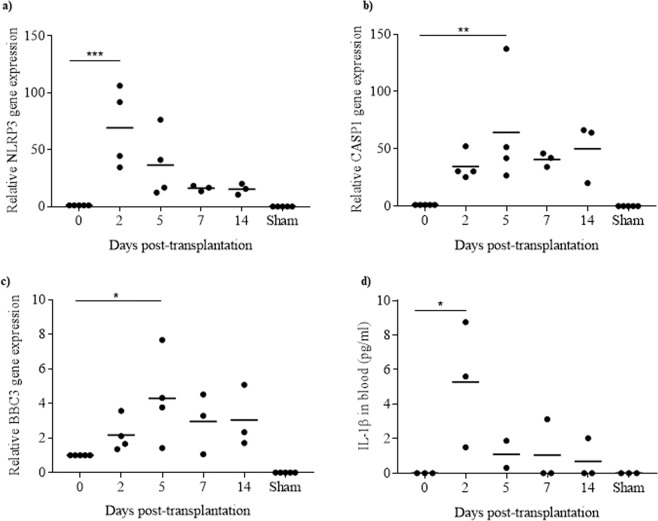
NLRP3 inflammasome is transiently activated in transplanted islets. Rat islets were transplanted under the kidney capsule of immunodeficient mice. Islets before transplantation (day 0) and islet grafts retrieved at different days post-transplantation were analysed for *NLRP3* (**a**), *CASP1* (**b**) and *BBC3* (**c**) expression by qRT-PCR; gene expressions in kidney capsule tissue (Sham) were also analysed. At the same time intervals, IL-1β released in the blood was quantified by ELISA (**d**) in transplanted or Sham-operated animals. N = 5 (**a–c**) and N = 3 (**d**). *p < 0.05, **p < 0.01, ***p < 0.001.

To determine whether NLRP3 inflammasome is activated in transplanted islets, rat islets were transplanted under the kidney capsule of immunodeficient mice and, at different times post-transplantation, blood was collected for IL-1β quantification and the graft harvested for *NLRP3, CASP1* and *BBC3* expression analysis. When compared to isolated islets before transplantation (d0), expression of *NLRP3* markedly increased after transplantation, with a peak at day 2 (Fig. 1a). Expression of *CASP1* significantly increased from day 0 to day 5, and then decreased to lower levels at days 7 and 14 (Fig. 1b). Expression of pro-apoptotic gene *BBC3* increased until day 5, and then tended to decrease at days 7 and 14 (Fig. 1c). *NLRP3, CASP1* and *BBC3* were undetectable in sham-operated mice (Fig. 1a–c). IL-1β concentration in sera was significantly higher 2 days after transplantation, and then decreased from day 5 on. IL-1β was undetectable in the sera of sham-operated mice (Fig. 1d).

Taken together, these results show that, after the first days following islet transplantation, NLRP3 inflammasome is activated in the graft.

### NLRP3 inflammasome is activated in response to hypoxia

**Figure 2:**
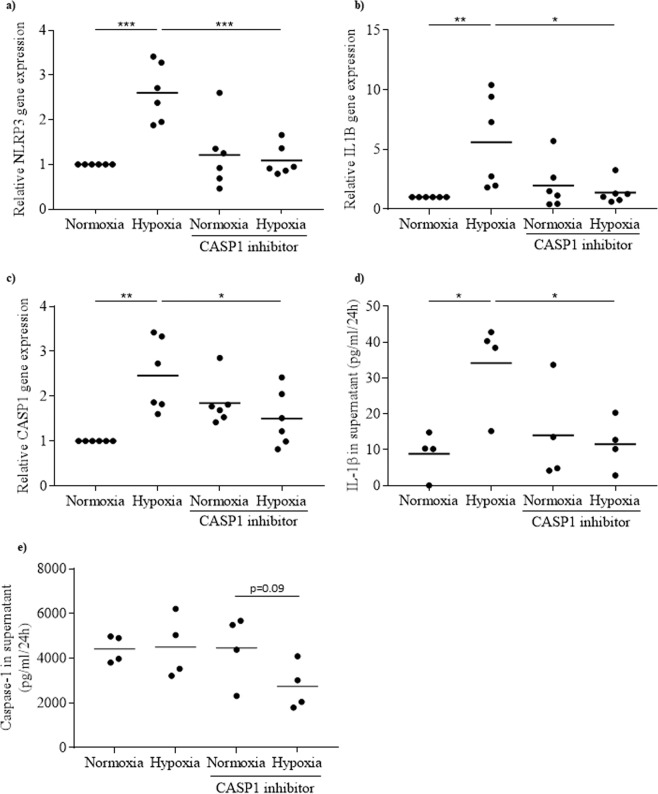
NLRP3 inflammasome is activated in response to hypoxia in rat islets. Rat islets were incubated either under normoxia or hypoxia for 24 h, in the absence or presence of CASP1 inhibitor. *NLRP3* (**a**), *IL1B* (**b**) and *CASP1* (**c**) were quantified by qRT-PCR. IL-1β **(d**) and caspase-1 (**e**) released in the supernatant were quantified by ELISA. N = 6 (**a–c**) and N = 4 (**d,e**). *p < 0.05, **p < 0.01, ***p < 0.001.

To assess whether rat islet NLRP3 inflammasome is activated in response to hypoxia, rat islets were incubated either under normoxia (control, 21% O_2_) or hypoxia (1% O_2_) for 24 h, and expressions of *NLRP3*, *IL1B* and *CASP1* analysed by real-time quantitative PCR. We observed that expressions of *NLRP3*, *IL1B* and *CASP1* increased 2.6 ± 0.3, 5.6 ± 1.6 and 2.5 ± 0.3 fold, respectively, (Fig. 2a–c) in response to hypoxia. The inhibitor of caspase-1 (CASP1 inhibitor), which blocks NLRP3 inflammasome activation and the maturation of IL-1β, significantly attenuated the hypoxia-induced expression of these genes (Fig. 2a–c). However, analysis of NLRP3 by immunoblot did not show an increase in NLRP3 in response to hypoxia. Therefore, no positive correlation was observed between NLRP3 gene and protein (Supplementary Fig. S1). The amount of IL-1β secreted in response to hypoxia increased compared to control conditions (Fig. 2d). Furthermore, the effect of hypoxia on IL-1β secretion was totally prevented by the presence of CASP1 inhibitor (Fig. 2d). Finally, caspase-1 released into the supernatant was prevented by CASP1 inhibitor (Fig. 2e).

Overall, these results show that NLRP3 inflammasome is activated by hypoxia and involved in IL-1β secretion induced by hypoxia in isolated rat islets.

### Activation of NLRP3 inflammasome in response to hypoxia is mediated by oxidative stress

**Figure 3:**
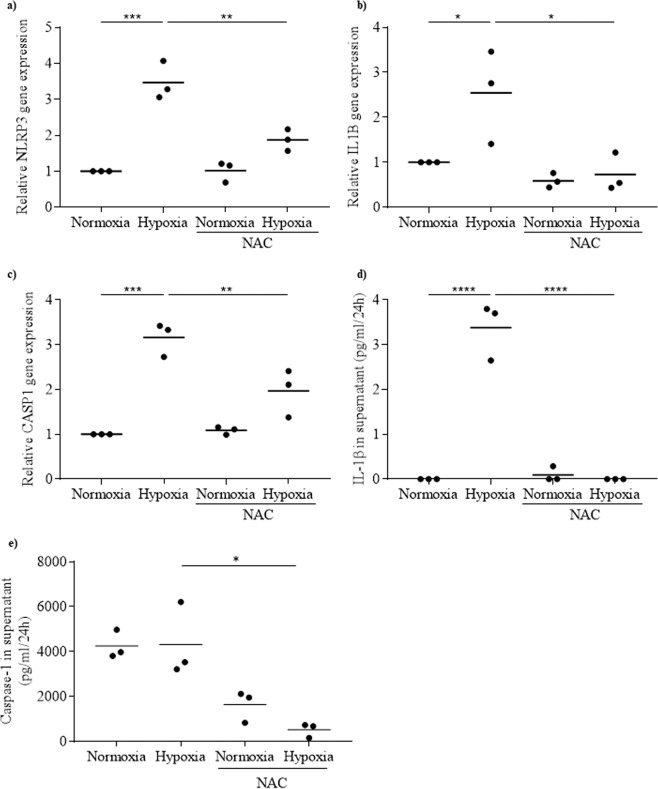
Activation of NLRP3 inflammasome by hypoxia in rat islets is mediated by oxidative stress. Rat islets were incubated either under normoxia or hypoxia for 24 h, in the absence or presence of NAC. *NLRP3* (**a**), *IL1B* (**b**) and *CASP1* (**c**) were quantified by qRT-PCR. IL-1β (**d**) and caspase-1 (**e**) released in the supernatant were quantified by ELISA. N = 3; *p < 0.05, **p < 0.01, ***p < 0.001, ****p < 0.0001.

To determine whether oxidative stress induced by hypoxia is involved in NLRP3 inflammasome activation, rat islets were incubated either under normoxic or hypoxic conditions for 24 h in the presence or absence of the stress oxidative inhibitor N-acetylcysteine (NAC). As shown above, hypoxia increased *NLRP3*, *IL1B*, *CASP1* expressions and IL-1β secretion (Fig. 3). Under normoxia, the presence of NAC did not affect the expression of these genes or IL-1β secretion (Fig. 3). By contrast, under hypoxia, NAC attenuated the increased expressions of *NLRP3* and *CASP1*, and prevented increased expression of *IL1B* and increased secretion of IL-1β and caspase-1 (Fig. 3). These results indicate that the activation of NLRP3 inflammasome by hypoxia is mediated by oxidative stress.

### NLRP3 inflammasome inhibition does not affect rat islet cell death induced by hypoxia

**Figure 4:**
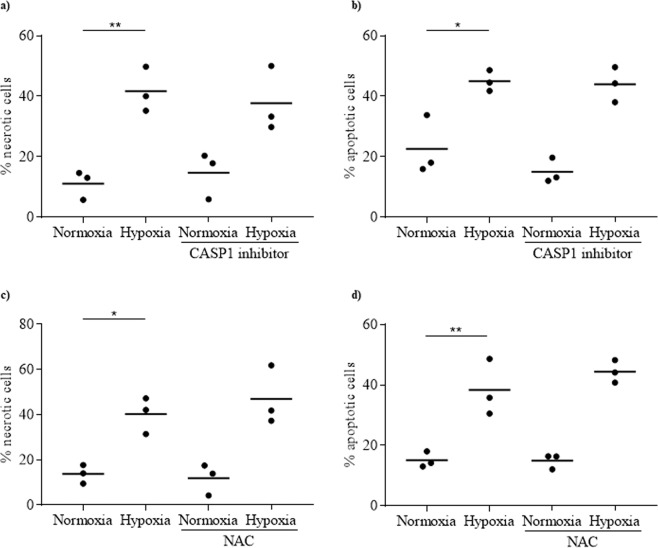
NLRP3 inflammasome is not involved in rat islet death induced by hypoxia. Rat islets were incubated either under normoxia or hypoxia for 24 h, in the absence or presence of CASP1 inhibitor or NAC. Necrosis (**a,c**) and apoptosis (**b,d**) were evaluated by propidium iodide and annexin V staining, respectively. N = 3; *p < 0.05, **p < 0.01.

**Figure 5:**
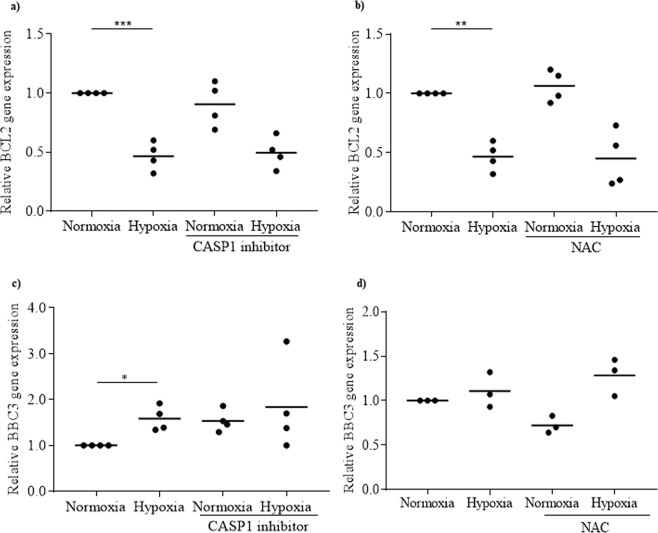
NLRP3 inflammasome do not affect apoptosis-related genes induced by hypoxia. Rat islets were incubated either under normoxia or hypoxia for 24 h, in the absence or presence of CASP1 inhibitor or NAC. *BCL2* (**a,b**) and *BBC3* (**c,d**) were quantified by qRT-PCR. N = 4; *p < 0.05, **p < 0.01, ***p < 0.001.

To determine whether NLRP3 inflammasome plays a role in rat islet cell death induced by hypoxia, NLRP3 inflammasome was inhibited by CASP1 inhibitor and any consequent apoptosis and necrosis were analysed by flow cytometry. As expected, hypoxia induced both necrosis (Fig. 4a) and apoptosis (Fig. 4b) in rat islet cells. However, neither necrosis nor apoptosis were prevented by CASP1 inhibitor, suggesting that NLRP3 inflammasome is not involved in rat islet cell death. When islet cell death induced by hypoxia was analysed in the presence of NAC, similar results were observed: neither necrosis (Fig. 4c) nor apoptosis (Fig. 4d) induced by hypoxia were prevented. Moreover, the inhibition of NLRP3 inflammasome by CASP1 inhibitor or NAC did not prevent the hypoxia-induced decrease of the anti-apoptotic gene *BCL2* expression (Fig. 5a,b) and the increase of the pro-apoptotic gene BBC3 (Fig. 5c,d). Taken together, these results demonstrate that NLRP3 inflammasome is not involved in rat islet cell death induced by hypoxia.

## Discussion

Hypoxia in islet transplantation is an inevitable phenomenon, which negatively affects the outcome of the graft^20,21^. Different actions are proposed to reduce this phenomenon such as decreasing the cold ischemia time, improving pancreas and islet preservation and oxygenation, and reducing the islet storage time before transplantation^22–24^. Another possible but not yet used approach would be the pharmacological targeting of the cellular pathways or processes activated by hypoxia. A prerequisite to developing this approach is a better understanding of the molecular mechanisms activated by hypoxia in islets. In a previous work^11^, we showed that hypoxia activates NLRP3 inflammasome in human islets. This multiprotein complex is involved in the maturation and production of the pro-inflammatory cytokine IL-1β. Since IL-1β is known to play a pivotal role in islet cell destruction after islet transplantation^14,25,26^, we thought to further investigate the expression of NLRP3 inflammasome in grafted rat islets, as well as the process activated in response to hypoxia. Consequently, we showed that islet expression of *NLRP3* and *CASP1* and IL-1β release increased shortly after islet transplantation. This is the first demonstration that NLRP3 inflammasome is activated in transplanted islets. Notably, the difference of *CASP1* expression between days 2 and 5 is not significant. The sustained expression of *CASP1* after day 2 suggests that caspase-1 may have another role beyond its involvement in inflammasome activation.

Our results are consistent with studies showing an increased production of IL-1β after islet transplantation^15^ and an activation of NLRP3 inflammasome during early insults, including ischemia/reperfusion injury, of transplanted solid organs^27^. We also found an increased expression of *BBC3* after islet transplantation. BBC3, also known as PUMA (p53 upregulated modulator of apoptosis), is a pro-apoptotic gene. Thus, activation of BBC3 is consistent with the hypothesis that the activation of apoptosis pathways in transplanted islets could result in graft dysfunction and/or rejection.

The activation of NLRP3 inflammasome following islet transplantation could be due to hypoxia. After 24 h incubation under hypoxia, *NLRP3*, *CASP1* and *IL-1β* expressions were increased in rat islets and released IL-1β was 3.8-fold higher than compared to control. However, no positive correlation was observed between NLRP3 gene and protein. A possible explanation for this result is that the increase in NLRP3 could be transient and NLRP3 could be rapidly degraded through the ubiquitin proteasome system. Hypoxia could induce ubiquitination of different proteins, modifying their function. It has been shown that the ubiquitination of NLRP3 protein induces its degradation^28^. Interestingly, inhibition of NLRP3 inflammasome and IL-1β maturation by CASP1 inhibitor prevented the hypoxia-induced expression and production of IL-1β, indicating that activation of NLRP3 inflammasome is involved in this hypoxia effect. These results are in agreement with our previous work, which reports that hypoxia is an inducer of NLRP3 inflammasome in human islets^11^. Hypoxia is known to be linked to oxidative stress. In response to hypoxia the mitochondria generates and releases ROS involved in the stabilization of HIF-1α^29,30^. When rat islets under hypoxia were treated with the inhibitor of oxidative stress, NAC, hypoxia-induced expression of inflammasome genes was prevented and secretion of IL-1β was abolished. This demonstrates that hypoxia induces NLRP3 inflammasome in rat islets through oxidative stress. Many studies showed that ROS produced under oxidative stress activate NLRP3 inflammasome^9,16,31,32^. Whether ROS mediate the NLRP3 inflammasome activation in response to hypoxia remains to be determined. Another important result is the impossibility to prevent hypoxia-induced islet cell death by targeting NLRP3 inflammasome. NLRP3 inflammasome inhibition preventing hypoxia-induced islet cell death was suggested by a study showing that apoptosis is not induced by hypoxia in islets isolated from NLRP3-deficient mice^5^. In our study, we used CASP1 inhibitor, which blocks NLRP3 inflammasome activation, and NAC, which blocks NLRP3 expression via its inhibitory action on oxidative stress. Both agents were unable to prevent the adverse effect of hypoxia on islet cell viability. Similar results were obtained when glyburide instead of CASP1 inhibitor was used to block NLRP3 inflammasome^11,33^. The reason for the discrepancy between the results obtained with NLRP3-deficient mice and NLRP3 inhibitors is unknown. With regard to NAC, we must note that its inhibitory effect on *NLRP3* and *CASP1* expressions in response to hypoxia is only partial and may explain why oxidative stress inhibition with NAC is unable to prevent hypoxia-induced cell death.

Other signalling pathways, independent or upstream of NLRP3 inflammasome and oxidative stress, could be involved in islet cell death induced by hypoxia. Apoptosis of islet cells in response to hypoxia could be mediated by p53, a tumour suppressor, shown to interact with HIF-1α under hypoxic conditions^34^. Both HIF-1α and p53 are protected from degradation by the proteasome in response to hypoxia^35^. PUMA activation is known to be involved in p53-mediated apoptosis^36,37^ and, interestingly, we observed an increase in *PUMA* (*BBC3)* expression in rat islets incubated *in vitro* under hypoxia and transplanted to immunodeficient mice. Other studies reported that PUMA is involved in cell death in human and rodent islets exposed to various insults^38–43^. A correlation has also been reported between *PUMA* gene expression and dysfunction of the grafted human islets in immunodeficient mice^38^.

In conclusion, our results show that NLRP3 inflammasome in rat islets is transiently activated after transplantation and induced through oxidative stress *in vitro*. In our model, we hypothesize that hypoxia induces ROS production (through mitochondrial damage), which consequently activate NF-κB (first signal) and lead to the transcription and translation of pro-IL-1β and NLRP3^44,45^. The second signal is also provided by ROS, which may activate inflammasome through the TXNIP-NLRP3 axis^32^. Interestingly, we observed an increase in *TXNIP* gene in rat islets in response to hypoxia suggesting the involvement of TXNIP in inflammasome activation (Supplementary Fig. S2). To further investigate the impact of NLRP3 inhibition on transplant outcome, it will be interesting to evaluate the function and survival of the graft after islet transplantation in a diabetic mouse treated with a caspase-1 inhibitor (Belnacasan VX765 from Selleck). When used *in vivo*, Belnacasan VX765 prevents the development of inflammatory bowel disease^46^, ameliorates liver fibrosis^47^ and prevents the progression of asthma^48^. Finally, we showed that NRLP3 inflammasome inhibition does not protect islet cells against hypoxia. Other NLRP3-independent pathways activated by hypoxia should be considered as targets to prevent adverse effects of hypoxia in islet transplantation.

## Supplementary information

Supplementary information.
