# Supplementary material for: NLRP3 Inflammasome is Activated in Rat Pancreatic Islets by Transplantation and Hypoxia
Source: Sci Rep. 2020 Apr 24;10:7011. doi: 10.1038/s41598-020-64054-9 (PMC7181690; doi:10.1038/s41598-020-64054-9)
Supplement: Supplementary file 1 — Supplementary information. [file 41598_2020_64054_MOESM1_ESM.pdf]

Supplementary information

NLRP3 Inflammasome is Activated in Rat Pancreatic Islets by Transplantation and Hypoxia

Vanessa Lavallard, David Cottet-Dumoulin, Charles-Henri Wassmer, Caroline Rouget, Géraldine Parnaud, Estelle Brioudes, Fanny Lebreton, Kevin Bellofatto, Ekaterine Berishvili, Thierry Berney, Domenico Bosco

Cell Isolation and Transplantation Center, Department of Surgery, Faculty Diabetes Center, Geneva University, Hospitals and University of Geneva, Geneva, Switzerland

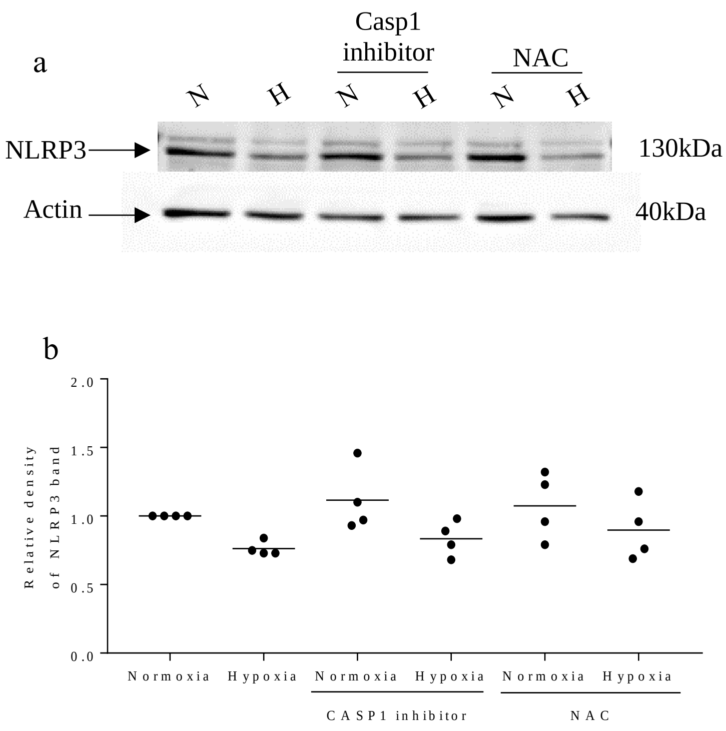

Figure S1 : Supplementary figure S1. NLRP3 protein expression in rat islets in response to hypoxia. Rat islets were incubated either under normoxia or hypoxia for 24 h, in the absence or presence of CASP1 inhibitor or NAC. Protein level of NLRP3 was analysed by immunoblot (a) and density of NLRP3 bands quantified (b). N=4. N, normoxia; H, hypoxia

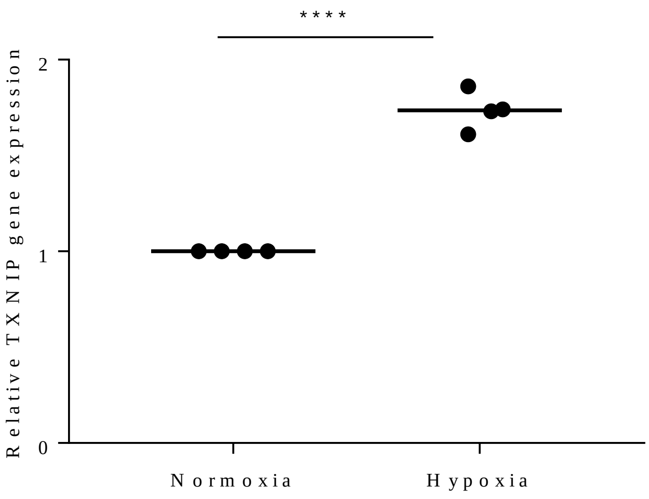

Figure S2. TXNIP gene expression in rat islets in response to hypoxia. Rat islets were incubated either under normoxia or hypoxia for 24 h. TXNIP was quantified by qRT-PCR. N=4. N, normoxia; H, hypoxia
